# Traumatic events and psychotic experiences: a nationally representative study in Thailand

**DOI:** 10.1017/S2045796021000172

**Published:** 2021-06-08

**Authors:** C. Kilian, S. Supanya, C. Probst, C. Morgan, T. Bärnighausen, P. Kittirattanapaiboon, P. Kwansanit, U. Reininghaus

**Affiliations:** 1 Institute of Clinical Psychology and Psychotherapy, Technical University of Dresden, Dresden, Germany; 2 Department of Mental Health, Somdet Chaopraya Institute of Psychiatry, Bangkok, Thailand; 3 Heidelberg Institute of Global Health (HIGH), Medical Faculty and University Hospital, Heidelberg University, Heidelberg, Germany; 4 Institute of Mental Health Policy Research, Centre for Addiction and Mental Health, Toronto, Ontario, Canada; 5 ESRC Centre for Society and Mental Health, Institute of Psychiatry, Psychology & Neuroscience, King's College London, London, UK; 6 National Institute for Health Research (NIHR) Mental Health Biomedical Research Centre at South London and Maudsley NHS Foundation Trust and King's College London, London, UK; 7 Department of Mental Health, Ministry of Public Health, Nonthaburi, Thailand; 8 Department of Public Mental Health, Central Institute of Mental Health, Medical Faculty Mannheim, University of Heidelberg, Mannheim, Germany

**Keywords:** Trauma, psychotic experiences, hallucination, delusion, Thailand

## Abstract

**Aims:**

Most research exploring the link between traumatic events and psychotic experiences has focused on either Australia, Europe or North America. In this study, we expand the existing knowledge to Thailand and investigate the impact of the type and the number of traumatic events on psychotic experiences in Thailand.

**Methods:**

We used data from the nationally representative 2013 Thai National Mental Health Survey (TNMHS), including questions on traumatic events and psychotic experiences. We regressed the lifetime experience of hallucinations or delusions against the following independent variables: the experience of any traumatic event during lifetime (dichotomous; hypothesis 1); the experience of either no traumatic event, one interpersonal, one unintentional or both interpersonal and unintentional traumatic events (categorical; hypothesis 2) and the number of traumatic events experienced during lifetime (categorical; hypothesis 3). We adjusted the regression models for sociodemographic indicators and psychiatric disorders, and considered survey weights.

**Results:**

About 6% (95% confidence interval: 4.9–7.0) of the respondents stated that they had either hallucinatory or delusional experiences during their lifetime. The risk of reporting such experiences was more than doubled as high among respondents who had experienced at least one traumatic event during their lifetime than among those who had not yet experienced one, with higher risks for interpersonal or multiple traumatic events. Our results further indicated an increase in the risk of psychotic experiences as the number of traumatic events increased, with up to an eight-fold higher risk for people exposed to five or more traumatic events in their lifetime, compared to those with no traumatic events.

**Conclusions:**

Individuals reporting interpersonal or multiple traumatic events face much higher risk of psychotic experiences. Effective and widely accessible secondary prevention programmes for people having experienced interpersonal or multiple traumatic events constitute a key intervention option.

## Introduction

Schizophrenia spectrum and other psychotic disorders are a heterogeneous group of mental disorders that are characterised by delusions, hallucinations, disorganised thinking, as well as diminished emotional expression and avolition (World Health Organization, 2019). They can severely affect a person's functioning in multiple areas of everyday life and are associated with substantial burden for individuals, carers and the wider society (Velthorst *et al*., 2017; Michalska da Rocha *et al*., 2018). Although almost 2 million people in Southeast Asia are estimated to meet the criteria for schizophrenia according to the International Classification of Diseases (ICD-10; World Health Organization, 1992) in 2016 (age-standardised point prevalence for Thailand, 2016: 0.28%, 95% confidence interval (CI): 0.24–0.31; Charlson *et al*., 2018), reliable epidemiological data remain scarce.

Psychotic experiences – i.e. delusional or hallucinatory experiences – are key symptoms of disorders within the schizophrenia spectrum and other psychotic disorders, although such experiences may also occur in individuals without a diagnosis of mental disorder (van Os *et al*., 2009; McGrath *et al*., 2015). The aetiology of psychotic experiences is complex and thought to imply an interplay of genetic vulnerability, neurobiological factors (prominently the activity of the hypothalamic–pituitary–adrenal axis and the dopamine system) and socio-environmental adversities (Morgan *et al*., 2010; Howes and Murray, 2014). Within this framework, stressful or traumatic life events seem to be an important component risk factor for psychotic experiences (Beards *et al*., 2013; Gibson *et al*., 2016; Mayo *et al*., 2017). Traumatic events in both childhood and adulthood have been found to be linked to the onset of psychotic experiences across multiple countries (Beards *et al*., 2013; Gibson *et al*., 2016; McGrath *et al*., 2017). In a recent multi-country study of cohort data (World Mental Health Survey), the occurrence of any traumatic event was associated with a three-fold higher risk of subsequent first onset of a psychotic experience compared with individuals who did not report traumatic events (McGrath *et al*., 2017). Furthermore, the risk of psychotic experiences increased in line with the number of traumatic events, suggesting a dose–response relationship. Such a dose–response relationship between the number of traumatic events and the likelihood of psychotic experiences has been already proposed in earlier studies (Scott *et al*., 2007; Shevlin *et al*., 2007; Beards *et al*., 2013). Furthermore, the few studies that examined different categories of traumatic events suggest that intrusive or interpersonal traumatic events are of particular importance for the development of psychoses compared with unintentional, non-interpersonal traumatic events (Raune *et al*., 2009; Beards *et al*., 2013; Solesvik *et al*., 2016; McGrath *et al*., 2017). Such interpersonal traumatic events are defined as events that are intentionally caused by other people (e.g. emotional abuse, neglect, abandonment, interpersonal violence and sexual assault), including collective violence (e.g. war and terror).

To date, the bulk of evidence on the association between traumatic events and psychotic experiences comes from countries such as Australia, Europe and North America, with a very limited number of countries from Southeast Asia such as Thailand. The objective of the current study was to address this research gap using data from the 2013 Thai National Mental Health Survey (TNMHS). According to the existing literature, we investigated the following hypotheses:

(1) Thai adults who have experienced at least one traumatic event are at higher risk of reporting psychotic experiences (i.e. hallucinations and delusional experiences).
(2) Interpersonal traumatic events are associated with higher risk of reporting psychotic experiences compared with unintentional, non-interpersonal traumatic events.
(3) There is a dose–response relationship between traumatic events and psychotic experiences, with higher risk for psychotic experiences with an increasing number of traumatic events.

## Methods

Data from the TNMHS in 2013 were used, a sub-nationally representative cross-sectional survey that adopted the methodology of the World Mental Health Survey Initiative (Kessler and Üstün, 2004). Stratified four-stage probability sampling was applied, including Thai residents (i.e. having a registered address in Thailand for at least past 3 months) aged 18 years or older. Survey weights were provided by the National Statistical Office of Thailand, accounting for the sample weight, non-responses, oversampling of women and elderly and a post-stratification calibration adjustment. Details of the methodology applied in the TNMHS have been published elsewhere (Kittirattanapaiboon *et al*., 2016).

The instrument used in the TNMHS was the Composite International Diagnostic Interview (CIDI), a fully standardised interview on mental disorders in accordance with the ICD-10 (Kessler and Üstün, 2004). The CIDI records symptoms of a broad range of mental disorders within varying reference periods. In order to apply the CIDI in Thailand, the survey was first translated by a research team of psychiatrists and linguists, and then piloted in clinical and general population samples. Interviews were conducted by trained interviewers who completed a 3-day workshop by instructors certified by the CIDI Training and Reference Centre, Institute of Social Research, University of Michigan.

In the current study, psychotic experiences were defined as reporting at least one experience of visual (‘Have you ever seen something that wasn't there that other people could not see?’) or auditory hallucinations (‘Have you ever heard any voices that other people said did not exist?’), or delusions such as thought insertion and/or withdrawal, mind control and/or passivity, ideas of references, plot to harm and/or being followed during lifetime, after excluding the possibility of dreaming or the influence of substances (‘not dreaming, not half-asleep, or not under the influence of alcohol or drugs’). Furthermore, respondents were asked whether they had ever experienced one or more traumatic events (for all traumatic events considered in this study, see Table 1). We distinguished between interpersonal (e.g. being kidnapped and sexual abuse) and unintentional, non-interpersonal traumatic events (e.g. traffic accidents and sever child illness) (Maercker, 2013). Traumatic events that were not specified or disclosed or that could not be clearly assigned to either category were not considered in analyses where the types of traumatic events were included (see Table 1). Lifetime post-traumatic stress disorder (PTSD), any past-year depression and any past-year anxiety disorder according to ICD-10 (World Health Organization, 1992) were considered as confounders (Isvoranu *et al*., 2017; Mayo *et al*., 2017), in addition to the following demographics and socioeconomic indicators (e.g. Fusar-Poli *et al*., 2017; Koenen *et al*., 2017): gender (women, men), age (continuous), educational attainment (primary or less, junior high, senior high, higher education) and employment status (employed, self-employed, unemployed).

**Table 1.** Classification of traumatic events into interpersonal and unintentional, non-interpersonal traumatic events

Interpersonal traumatic event	Unintentional traumatic event	Not specified
Beaten-up by caregiver	Automobile accident	Not disclosed
Beaten-up by someone else	Child with serious illness	Other traumatic event
Beaten-up by spouse or romantic partner	Life-threatening illness	Traumatic event of a loved one
Civilian in a warzone	Natural disaster	Witnessed death, dead body or someone seriously hurt
Civilian in region of terror	Other life-threatening accident	
Combat experiences	Toxic chemical exposure	
Kidnapped	Unexpected death of a loved one	
Man-made disaster	Unintentionally caused serious injury or death	
Mugged or threatened with weapon		
Purposely injured, tortured or killed someone		
Raped		
Refugee		
Relief worker in warzone		
Saw atrocities		
Sexually assaulted		
Stalked		
Witnessed physical fights at home		

### Statistical analysis

Poisson regression models with robust standard error estimation (Rojanaworarit and Wong, 2019) were used to examine the association between traumatic events and psychotic experiences. The outcome of interest was the occurrence of at least one psychotic experience during one's lifetime (dichotomous). As independent variable, the experience of at least one traumatic event was considered (dichotomous, hypothesis 1). In order to test hypothesis 2, the same modelling approach was applied considering a categorical independent variable distinguishing between persons who did not experience a traumatic event, experienced at least one unintentional traumatic event, at least one interpersonal traumatic event or experienced both an unintentional and interpersonal traumatic event (four levels). Individuals who did not report interpersonal or unintentional traumatic events, but another, unspecified traumatic event, were excluded in this analysis (4.4% missing). Finally, in order to examine a dose–response relationship (hypothesis 3), the number of traumatic events was taken into account as categorical independent variable with five levels (0, 1, 2, 3, 4 and 5 or more traumatic events).

In order to adjust the regression models, the following control variables were included consecutively: demographic variables (gender, age; model 1), socioeconomic indicators (educational attainment, employment status; model 2) and finally psychiatric disorders (fulfilling diagnostic criteria for PTSD at any time during lifetime, past-year depressive and past-year anxiety disorder; model 3). Additionally, an unadjusted model was used (model 0). Respondents with missing information on either the dependent, independent or control variables were excluded from analyses (*N* = 12). Survey weights were applied in all regression analyses. All analyses were carried out using Stata 15.1 (StataCorp, 2017).

### Ethical approval

The TNMHS was approved by the Ministry of Public Health's Committee for Mental Health and Psychiatric Research in Humans on 21 October 2013 with registration number 77/2556.

## Results

### Sample characteristics

A total of 4727 adults completed the interview (response rate: 74.3%), of which 4715 aged 44.1 years on average (standard deviation [s.d.]: 23.4 years) constituted the analytic sample (<1% missing). An overview of sociodemographic characteristics of the sample, the prevalence of psychotic experiences, the prevalence of reporting at least one traumatic event and the weighted percentage of persons fulfilling diagnostic criteria for mental disorders (i.e. PTSD diagnosis at any time during lifetime, past-year depressive disorder, past-year anxiety disorder) is displayed in Table 2.

**Table 2.** Overview of sample characteristics and prevalence of psychotic experiences, traumatic events and mental disorders within the study population (total *N* = 4715)

Variable	Total number	Prevalence (%)^a^	95% CI
Gender: women	3002	51.8	49.7–53.8
Educational attainment			
Primary or less education	2888	48.2	46.1–50.2
Junior high school	473	12.9	11.4–14.5
Senior high school	767	22.2	20.3–24.1
Tertiary education	587	16.8	15.2–18.6
Employment status			
Employed	1562	37.2	35.2–39.3
Self-employed	1908	38.4	36.4–40.4
Unemployed	1245	24.4	22.7–26.2
Any psychotic experience during lifetime			
Visual hallucinations	132	3.0	2.4–3,9
Auditory hallucinations	151	3.1	2.5–3.9
Delusional experiences	46	0.9	0.6–1.5
Traumatic events during lifetime^b^			
One traumatic event	1394	29.3	27.5–31.2
Unintentional traumatic event^c^	1502	31.9	30.0–33.9
Interpersonal traumatic event^c^	249	5.8	4.8–7.0
Unintentional and interpersonal traumatic event^c^	508	10.9	9.7–12.2
Mental disorders			
Diagnosis of PTSD (at any time during lifetime)	48	0.9	0.6–1.3
Past-year depressive disorder	42	0.7	0.4–1.0
Past-year anxiety disorder	73	1.3	1.0–1.8

a Survey weights applied to account for the population distribution.

b Numbers do not add up as 21.8% of the participants have experienced more than one traumatic event.

c *N* = 4506.

About 6% (95% CI: 4.9–7.0) of the respondents reported a psychotic experience during their lifetime, with hallucinatory experiences being more prevalent than delusional experiences. One in two respondents reported at least one traumatic event during their lifetime, with 12.8% (95% CI: 11.5–14.2) reporting two, 5.3% (95% CI: 4.5–6.4) three, 1.9% (95% CI: 1.5–2.5) four and 1.7% (95% CI: 1.3–2.3) five traumatic events. Unintentional, non-interpersonal traumatic event were reported more often than interpersonal traumatic events. On average, the first traumatic event was reported at the age of 26.1 years (s.d.: 19.5 years), with almost two-fifths reporting having been under the age of 18 (38.0%, 95% CI: 35.1–40.9; *N* = 2444, 1.2% missing). Almost 1% (0.9%, 95% CI: 0.6–1.3) of the respondents met the criteria for PTSD at any time during their lifetime.

### Traumatic events and psychotic experiences

Results of the Poisson regression models conducted for hypotheses testing are displayed in Table 3. Respondents reporting at least one traumatic event during their lifetime were around 2.3 times more likely to report a psychotic experience. Taking into account the type of traumatic events, the likelihood of reporting psychotic experiences was similar among respondents experiencing an unintentional traumatic event only and those individuals without any traumatic event. By contrast, respondents experiencing at least one interpersonal traumatic event were around 2.3 times more likely to report psychotic experiences during their lifetime (compared to those without traumatic events). Among those who reported both at least one unintentional and one interpersonal traumatic event, the risk of psychotic experiences was about 4.0 times higher than among those without traumatic event.

**Table 3.** Results of the Poisson regression models for the association of traumatic events and psychotic experiences for (1) any traumatic event during lifetime and (2) by type of traumatic event

	Model 0			Model 1			Model 2			Model 3		
	RR	*p*-value	95% CI	RR	*p*-value	95% CI	RR	*p*-value	95% CI	RR	*p*-value	95% CI
Independent variable: experiencing any traumatic events during lifetime												
No traumatic event	1.0			1.0			1.0			1.0		
Experiencing at least one traumatic event	2.4	<0.001	1.6–3.7	2.5	<0.001	1.6–3.8	2.5	<0.001	1.6–3.8	2.3	<0.001	1.5–3.5
Independent variable: type of traumatic event^a^												
No traumatic event	1.0			1.0			1.0			1.0		
Experiencing unintentional traumatic event only	1.6	0.073	1.0–2.5	1.6	0.058	1.0–2.5	1.6	0.050	1.0–2.5	1.5	0.073	1.0–2.4
Experiencing interpersonal traumatic event only	2.8	0.005	1.4–5.7	2.8	0.006	1.3–5.7	2.7	0.006	1.3–5.5	2.3	0.024	1.1–4.8
Experiencing both unintentional and interpersonal traumatic event	4.4	<0.001	2.7–7.2	4.6	<0.001	2.8–7.5	4.5	<0.001	2.8–7.3	4.0	<0.001	2.5–6.6

RR, risk ratios; CI, confidence interval.

Model 0: unadjusted model.

Model 1: adjusted for sex and age.

Model 2: adjusted for sex, age, educational attainment and employment status.

Model 3: adjusted for sex, age, educational attainment, employment status, PTSD diagnosis at any time during lifetime, past-year depressive disorder and past-year anxiety disorder.

a *N* = 4506; 4.4% missing values due to respondents reported an unspecified traumatic event.

**Fig. 1.**
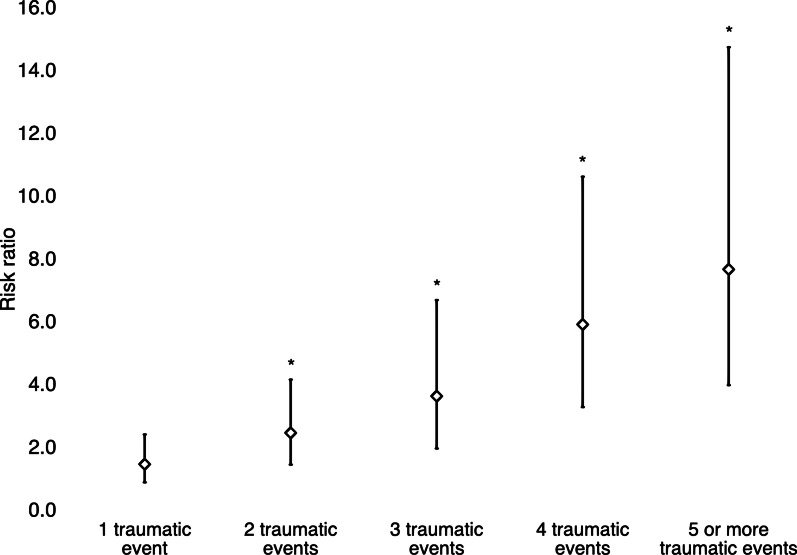
Dose–response relationship (risk ratios and 95% CIs) between the number of traumatic events and psychotic experiences. Reference category: no traumatic event. Model adjusted for sex, age, educational attainment, employment status, PTSD diagnosis at any time during lifetime, past-year depressive disorder and past-year anxiety disorder. *p < 0.001.

The risk of reporting psychotic experiences as a function of the number of traumatic events is shown in Fig. 1. The risk ratios of psychotic experiences in respondents who were exposed to a single traumatic event was not statistically significantly higher than for individuals who were not exposed to a traumatic event (*p* = 0.103). Beginning with two traumatic events, the risk of psychotic experiences increases with each additional event, with risk ratios of 2.5 (95% CI: 1.5–4.2), 3.7 (95% CI: 2.0–6.7) and 5.9 (95% CI: 3.3–10.7) for two, three and four traumatic events, respectively. Compared with respondents without traumatic event, those who reported five or more traumatic events had 7.7 (95% CI: 4.0–14.8) times higher risk of having at least one psychotic experience during their lifetime.

## Discussion

To the best of our knowledge, this is the first study investigating the link between traumatic events and psychotic experiences in the Thai population. In line with previous research, we found an increased risk for psychotic experiences in individuals who have suffered from traumatic events (Scott *et al*., 2007; Shevlin *et al*., 2007; Raune *et al*., 2009; Beards *et al*., 2013; Solesvik *et al*., 2016; Mayo *et al*., 2017; McGrath *et al*., 2017). Moreover, among individuals who were exposed to either interpersonal or multiple traumatic events (i.e. more than one event), the risk of psychotic experiences was particularly high compared with those who reported a single unintentional or no traumatic event.

The risk of psychotic experiences increased with the number of traumatic events, pointing to a dose–response relationship which is in line with evidence from previous research (Scott *et al*., 2007; Shevlin *et al*., 2007; Beards *et al*., 2013; Morgan and Gayer-Anderson, 2016; Croft *et al*., 2019). There was no strong evidence of an increased likelihood of psychotic experiences when only a single traumatic event was reported unless this was an interpersonal event. These findings lead us to conclude – without assuming any causality – that the risk of psychotic experiences is generally not increased when a person is exposed to a single unintentional traumatic event. However, when a single traumatic event involved an interpersonal situation, the likelihood of psychotic experiences is increased. According to Morgan and colleagues (Morgan *et al*., 2010; Morgan and Gayer-Anderson, 2016), adversities in child- and adulthood are key factors in the socio-developmental pathway to psychosis. Exposure to trauma is supposed to interplay with the genetic susceptibility and the dopaminergic system of an individual and to affect its perceptions and cognitions, leading to initial manifestations of a psychosis in some people. Intrusive life events, including interpersonal traumatic events, are proposed to be specifically linked to psychosis (Morgan and Gayer-Anderson, 2016), which is supported by our findings.

Some limitations have to be taken into account. First, the cross-sectional design of our study does not allow for conclusions about causality. Second, since the TNMHS was conducted in 2013, more research is needed using more recent databases, including other countries in this region, and taking into account longitudinal study designs. Third, psychotic experiences constitute only one symptom of schizophrenia spectrum and other psychotic disorders and may not in all cases lead to clinically meaningful impairment of functioning. Additionally, the TNMHS did not include questions on family history of psychotic experiences, which would have been a key variable given the large genetic component in schizophrenia and other psychotic disorders (e.g. Chou *et al*., 2017; Zwicker *et al*., 2018). Fourth, the regression of the type of traumatic event (hypothesis 2) is not completely independent of the dose–response regression (hypothesis 3), since in the former analysis we have considered one category in which both types of traumatic events are present (i.e. interpersonal and unintended events), i.e. a minimum of two events, which is equivalent to the experience of two or more traumatic events from the dose–response analysis. In this context, it must be taken into account that no information was available on whether the traumatic events were experienced on one or several independent situations. For example, it could have happened that an unexpected death of a loved one (traumatic event 1) occurred in a car accident in which the respondent was personally involved (traumatic event 2). However, we consider the impact of these restrictions on the relevance of our study results to be marginal.

Based on the strong link between traumatic events and psychotic experiences, some researchers call for a distinction to be made between trauma-related psychotic experiences and such experiences in people who were not exposed to trauma (Hardy, 2017; Wearne *et al*., 2020), with major implications for diagnosis and treatment (Alameda *et al*., 2020). Research investigating potential causal pathways of the association between trauma and psychosis found dissociation – a symptom of post-traumatic stress – to be associated with psychotic experiences (Longden *et al*., 2020; Wearne *et al*., 2020) and may be a putative mechanism underlying the association (Perona-Garcelán *et al*., 2012; Varese *et al*., 2012; Schalinski *et al*., 2019; Alameda *et al*., 2020). Additionally, trauma-related beliefs as well as negative cognitive schemas may serve as such putative mechanisms (Alameda *et al*., 2020; Hardy *et al*., 2020). However, the multitude of research undertaken so far is limited by its narrow focus on interpersonal traumatic events and/or childhood adversities, hence studying only specific types of traumatic events, not allowing for a systematic comparison between the risks related to different types of traumatic events. Yet, studies that considered the type (i.e. interpersonal and/or unintentional traumatic events) and the timing of traumatic events (e.g. trauma exposure in childhood, adolescence or adulthood) indicate differences in risk for psychotic experiences across these two aspects (i.e. type and timing of traumatic events; Shevlin *et al*., 2007; Schroeder *et al*., 2016; Croft *et al*., 2019; Schalinski *et al*., 2019). Our findings concur with previous results and provide further insights into the role of interpersonal trauma and the exposure to multiple traumatic events, which seems to be independent of the type of trauma. Given our study's limitations, we may recommend the following research opportunities for future studies: exploring different types and the timing of traumatic events; distinguishing between traumatic events, in which the person interviewed was either a victim, a perpetrator or both a victim and a perpetrator – a distinction which was not possible in our study; and investigating the trauma–psychosis relationship using a longitudinal study design.

Our findings emphasise the close link between traumatic events and psychotic experiences and extend the existing knowledge to the region of Southeast Asia. Experiencing either an interpersonal trauma or from multiple traumatic events were particularly associated with reporting psychotic experiences during lifetime. In Thailand, almost one in five individuals reported having experienced either an interpersonal trauma or multiple traumatic events in their lifetime, and two in five were minors at the time of first trauma exposure. With a total population of 69.8 million inhabitants in Thailand in 2020 (United Nations, Department of Economic and Social Affairs, Population Division, 2020), about 13 million people would be potentially at risk during their lifetimes. These numbers exemplify the need for effective secondary prevention programmes for people who have experienced interpersonal or multiple traumatic events, particularly for those with a genetic predisposition for psychosis (Fusar-Poli *et al*., 2017). According to the World Health Organisation's recommendations within the Mental Health Gap Action Programme to reduce the treatment gap for mental disorders in low- and middle-income countries (World Health Organization *et al*., 2008, 2015), psychological support should be provided in terms of psychological first aid for those with acute stress following traumatic events (Dua *et al*., 2011: 20). Furthermore, prevention (and intervention) programmes may target trauma-related beliefs, negative cognitive schemas or dissociative symptoms, which have been previously found to be putative mechanisms for trauma-related psychosis and distinct from those in non-trauma-exposed persons with psychosis (Alameda *et al*., 2020; Hardy *et al*., 2020). Secondary prevention programmes may consider mobile apps or other remote and electronic interventions as an option, as they are easily accessible and preliminary evidence of favourable outcomes and high acceptance in the patient population exists (Vaidyam *et al*., 2019).

## Conclusion

This study was the first to investigate the association between traumatic events and psychotic experiences in a Thai population. We found the experience of interpersonal or multiple traumatic events to be associated with a substantially elevated likelihood of reporting hallucinations or delusions during lifetime. Within our sample, a total of 6% reported having had psychotic experiences at least once in their lives, and one in five respondents stated having experienced interpersonal or multiple traumatic events. The high lifetime prevalence of traumatic and psychotic experiences in the Thai population highlights the need for effective and widely accessible secondary prevention programmes to reduce post-traumatic stress in affected people.

## Availability of data and materials

The dataset supporting the conclusions of this paper will be made available upon request to the Bureau of Mental Health Academic Affairs, Department of Mental Health, Ministry of Public Health: dmh.bmha@gmail.com. Syntax used in the statistical analyses can be obtained upon request to the corresponding author.
